# (*R*,*S*_P_)-1-Diphenyl­phosphino-2-(1-ethoxy­ethyl)ferrocene

**DOI:** 10.1107/S1600536808033618

**Published:** 2008-10-22

**Authors:** Hao Yuan, Zhi-Ming Zhou

**Affiliations:** a School of Chemical Engineering and the Enviroment, Beijing Institute of Technology, Beijing 100081, People’s Republic of China

## Abstract

In the crystal structure of the title compound, [Fe(C_5_H_5_)(C_21_H_22_OP)], the cyclo­penta­dienyl (Cp) rings are almost parallel and are essentially eclipsed. The absolute configuration was determined as *S* for the planar and *R* for the central chirality.

## Related literature

For background to ferrocene derivatives applied as catalysts, see: Blaser & Schmidt (2004 ▶); Gomez Arrayas *et al.* (2006 ▶); Hayashi *et al.* (1988 ▶); Ohmura *et al.* (1995 ▶); Ojima (2000 ▶). For the structures of closely related compounds, see: Jin *et al.* (2004 ▶); Cheelama & Knochel (2007 ▶); Podlaha *et al.* (1996 ▶).
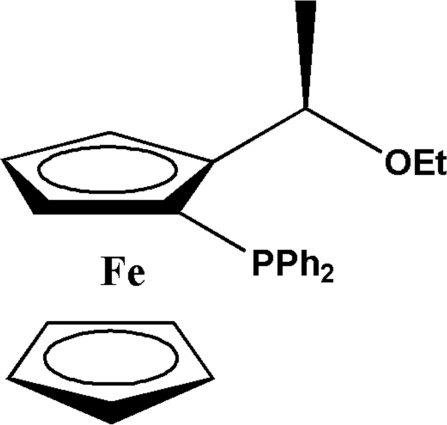


## Experimental

### 

#### Crystal data

[Fe(C_5_H_5_)(C_21_H_22_OP)]
*M* *_r_* = 442.30
Orthorhombic, 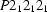

*a* = 11.003 (2) Å
*b* = 12.191 (2) Å
*c* = 16.599 (3) Å
*V* = 2226.6 (8) Å^3^
*Z* = 4
Mo *K*α radiation
μ = 0.76 mm^−1^
*T* = 113 (2) K
0.12 × 0.10 × 0.08 mm

#### Data collection

Rigaku Saturn diffractometer
Absorption correction: multi-scan (*CrystalClear*; Rigaku, 2001 ▶) *T*_min_ = 0.914, *T*_max_ = 0.942
22928 measured reflections
3929 independent reflections
3847 reflections with *I* > 2σ(*I*)
*R*_int_ = 0.050

#### Refinement

*R*[*F* ^2^ > 2σ(*F* ^2^)] = 0.026
*wR*(*F* ^2^) = 0.062
*S* = 1.05
3929 reflections
262 parameters
H-atom parameters constrained
Δρ_max_ = 0.18 e Å^−3^
Δρ_min_ = −0.28 e Å^−3^
Absolute structure: Flack (1983 ▶), with 1688 Friedel pairs
Flack parameter: 0.019 (12)

### 

Data collection: *CrystalClear* (Rigaku, 2001 ▶); cell refinement: *CrystalClear*; data reduction: *CrystalClear*; program(s) used to solve structure: *SHELXS97* (Sheldrick, 2008 ▶); program(s) used to refine structure: *SHELXL97* (Sheldrick, 2008 ▶); molecular graphics: *SHELXTL* (Sheldrick, 2008 ▶); software used to prepare material for publication: *SHELXTL*.

## Supplementary Material

Crystal structure: contains datablocks global, I. DOI: 10.1107/S1600536808033618/nc2112sup1.cif

Structure factors: contains datablocks I. DOI: 10.1107/S1600536808033618/nc2112Isup2.hkl

Additional supplementary materials:  crystallographic information; 3D view; checkCIF report

## Figures and Tables

**Fig. 1.**
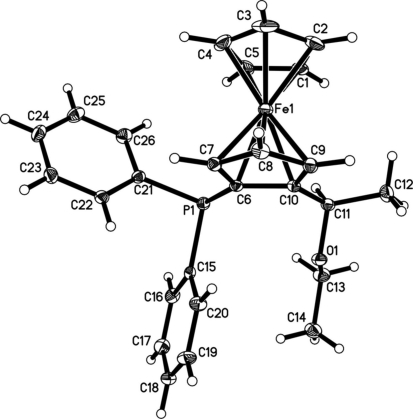
A molecular view of the title compound, showing the atom-labelling scheme. Displacement ellipsoids are draw at the 30% probability level.

## supplementary crystallographic information

### Comment

Asymmetric metal catalysis is one of the most active areas in modern organic chemistry, and considerable efforts have been made to the development of novel ligands for catalytic asymmetric transformations (Ojima, 2000). In this context, ferrocene-based ligands incorporating both chirality are very important (Gomez Arrayas *et al.*, 2006) and some of them have already been applied in industrial processes because of their stability, low price and unique structure (Blaser & Schmidt, 2004).

(*S*,*R*_p_)-1-(diphenylphosphino)-2-(1-ethoxyethyl)-ferrocene, the enantiomorph of title compound, has been used to synthesize 1,1'-binaphthyls *via* asymmetric Ni-catalysed Grignard cross-coupling with up to 68% ee, and the (*S*,*R*_p_)-1-(diphenylphosphino)-2-(1-methoxyethyl)-ferrocene provided axially chiral binaphthalenes in enantioselectivities up to 95% ee (Hayashi *et al.*, 1988). In addition, the (*R*,*S*_p_)-1-(diphenylphosphino)-2-(1-ethoxyethyl)-ferrocene was also used in asymmetric hydrosilyation (Ohmura *et al.*, 1995).

The Fe—C bond distances within the ferrocene group are in the range of 2.038 (2)–2.050 (2) Å for the unsubstituted cyclopentadienyl (Cp) ring [C1–C5] and 2.025 (2)–2.046 (2) Å for the substituted Cp ring [C6–C10]. The Cp rings are almost parallel, the dihedral angle between the Cp ring planes is 1.80 (10)°. The Cp rings are essentially eclipsed and the Fe–centroid distances are 1.654 (9) (*Cg*1) and 1.639 (9) Å (*Cg*2) with *Cg*1 and *Cg*2 are the centroids of the [C1–C5] and [C6–C10] rings. The [*Cg*1—Fe1—*Cg*2] angle is 178.60 (18)°. The C11 atom is almost in the plane of their carrier Cp ring, while the P1 atom is tilted slightly out of the plane by 0.102 (10) Å.

The two phenyl rings are oriented almost perpendicular, with a dihedral angle of 90.90 (10)°. The O1—C11 and C10—C11 bonds lengths are in agreement with those in the related complex 1-(1-Ferrocenyl-1-methoxy-3-phenyl-2-propyl)-1*H*-1,2,4-triazole (Jin *et al.*, 2004) and the geometric parameters of the PPh_2_ group are in agreement with those in the similar structure 1-carboxy-1'-(diphenylphosphino)-ferrocene (Podlaha *et al.*, 1996).

The title compound has both central chirality and planar chirality with the configuration of C11 atom being *R*, and the configuration of planar chirality being *S*.

### Experimental

The title compound was prepared from (*R*,*S*_p_)-1-[1-(acetyloxy)ethyl]-2-(diphenylphosphino)-ferrocene according to literature procedures (Hayashi *et al.*, 1988). Single crystals of the title compound suitable for X-ray diffraction analysis were obtained by slow evaporation of a hexane solution.

### Refinement

All H atoms were positioned with idealized geometry with C—H = 0.93 (aromatic), 0.96 (methyl), 0.97 (methylene) or 0.98 Å (cyclopentadienyl and Cp—CH) and were refined isotropic with *U*_iso_(H) = 1.2*U*_eq_(C) or 1.5*U*_eq_(methyl C) using a riding model. The absolute structure was determined on the basis of 1688 Friedel pairs.

### Crystal data

**Table:**

[Fe(C_5_H_5_)(C_21_H_22_OP)]	*F*(000) = 928
*M**_r_* = 442.30	*D*_x_ = 1.319 Mg m^−^^3^
Orthorhombic, *P*2_1_2_1_2_1_	Mo *K*α radiation, λ = 0.71073 Å
Hall symbol: P 2ac 2ab	Cell parameters from 7176 reflections
*a* = 11.003 (2) Å	θ = 2.2–27.9°
*b* = 12.191 (2) Å	µ = 0.76 mm^−^^1^
*c* = 16.599 (3) Å	*T* = 113 K
*V* = 2226.6 (8) Å^3^	Block, red
*Z* = 4	0.12 × 0.10 × 0.08 mm

### Data collection

**Table:**

Rigaku Saturn diffractometer	3929 independent reflections
Radiation source: rotating anode	3847 reflections with *I* > 2σ(*I*)
confocal	*R*_int_ = 0.050
ω scans	θ_max_ = 25.0°, θ_min_ = 2.1°
Absorption correction: multi-scan (CrystalClear; Rigaku, 2001)	*h* = −13→12
*T*_min_ = 0.914, *T*_max_ = 0.942	*k* = −14→14
22928 measured reflections	*l* = −19→19

### Refinement

**Table:**

Refinement on *F*^2^	Secondary atom site location: difference Fourier map
Least-squares matrix: full	Hydrogen site location: inferred from neighbouring sites
*R*[*F*^2^ > 2σ(*F*^2^)] = 0.026	H-atom parameters constrained
*wR*(*F*^2^) = 0.062	*w* = 1/[σ^2^(*F*_o_^2^) + (0.0301*P*)^2^ + 0.2869*P*] where *P* = (*F*_o_^2^ + 2*F*_c_^2^)/3
*S* = 1.05	(Δ/σ)_max_ = 0.002
3929 reflections	Δρ_max_ = 0.18 e Å^−^^3^
262 parameters	Δρ_min_ = −0.28 e Å^−^^3^
0 restraints	Absolute structure: Flack (1983), with 1688 Friedel pairs
Primary atom site location: structure-invariant direct methods	Flack parameter: 0.019 (12)

### Special details

**Table:**

Geometry. All e.s.d.'s (except the e.s.d. in the dihedral angle between two l.s. planes) are estimated using the full covariance matrix. The cell e.s.d.'s are taken into account individually in the estimation of e.s.d.'s in distances, angles and torsion angles; correlations between e.s.d.'s in cell parameters are only used when they are defined by crystal symmetry. An approximate (isotropic) treatment of cell e.s.d.'s is used for estimating e.s.d.'s involving l.s. planes.
Refinement. Refinement of *F*^2^ against ALL reflections. The weighted *R*-factor *wR* and goodness of fit *S* are based on *F*^2^, conventional *R*-factors *R* are based on *F*, with *F* set to zero for negative *F*^2^. The threshold expression of *F*^2^ > σ(*F*^2^) is used only for calculating *R*-factors(gt) *etc*. and is not relevant to the choice of reflections for refinement. *R*-factors based on *F*^2^ are statistically about twice as large as those based on *F*, and *R*- factors based on ALL data will be even larger.

### Fractional atomic coordinates and isotropic or equivalent isotropic displacement parameters (Å^2^)

**Table:**

	*x*	*y*	*z*	*U*_iso_*/*U*_eq_	
Fe1	−0.03864 (3)	0.77799 (2)	0.905322 (18)	0.02169 (9)	
P1	0.07707 (4)	0.98992 (4)	1.01327 (3)	0.01930 (12)	
O1	0.18641 (12)	1.04499 (11)	0.82901 (9)	0.0230 (3)	
C1	0.09790 (19)	0.66545 (15)	0.91286 (14)	0.0272 (5)	
H1A	0.1815	0.6758	0.8941	0.033*	
C2	0.0010 (2)	0.62243 (18)	0.86702 (16)	0.0358 (6)	
H2A	0.0055	0.5980	0.8108	0.043*	
C3	−0.1039 (2)	0.62123 (19)	0.9163 (2)	0.0513 (8)	
H3A	−0.1850	0.5958	0.9004	0.062*	
C4	−0.0708 (3)	0.6634 (2)	0.99241 (18)	0.0517 (8)	
H4A	−0.1254	0.6724	1.0386	0.062*	
C5	0.0538 (2)	0.69041 (18)	0.99089 (14)	0.0373 (6)	
H5A	0.1011	0.7209	1.0356	0.045*	
C6	−0.01527 (17)	0.94062 (15)	0.93016 (11)	0.0178 (4)	
C7	−0.14254 (18)	0.91375 (16)	0.92785 (12)	0.0210 (4)	
H7A	−0.2004	0.9224	0.9723	0.025*	
C8	−0.16938 (19)	0.87040 (17)	0.84996 (13)	0.0260 (5)	
H8A	−0.2489	0.8442	0.8315	0.031*	
C9	−0.06047 (19)	0.87097 (17)	0.80413 (12)	0.0230 (4)	
H9A	−0.0519	0.8449	0.7486	0.028*	
C10	0.03465 (19)	0.91267 (15)	0.85299 (11)	0.0198 (4)	
C11	0.16655 (19)	0.92877 (16)	0.83152 (12)	0.0204 (4)	
H11A	0.2167	0.8975	0.8746	0.024*	
C12	0.2039 (2)	0.87699 (19)	0.75210 (13)	0.0306 (5)	
H12A	0.2887	0.8903	0.7428	0.046*	
H12B	0.1572	0.9086	0.7091	0.046*	
H12C	0.1894	0.7994	0.7542	0.046*	
C13	0.3051 (2)	1.07659 (18)	0.85426 (15)	0.0291 (5)	
H13A	0.3658	1.0419	0.8204	0.035*	
H13B	0.3188	1.0538	0.9095	0.035*	
C14	0.3145 (2)	1.19937 (17)	0.84759 (15)	0.0334 (5)	
H14A	0.3939	1.2225	0.8645	0.050*	
H14B	0.2541	1.2329	0.8813	0.050*	
H14C	0.3014	1.2210	0.7927	0.050*	
C15	0.06768 (17)	1.14024 (16)	1.00256 (11)	0.0189 (4)	
C16	0.1436 (2)	1.20172 (18)	1.05214 (12)	0.0253 (5)	
H16A	0.1945	1.1661	1.0885	0.030*	
C17	0.1443 (2)	1.31563 (18)	1.04809 (13)	0.0308 (5)	
H17A	0.1950	1.3558	1.0819	0.037*	
C18	0.0702 (2)	1.36905 (18)	0.99405 (13)	0.0300 (5)	
H18A	0.0702	1.4452	0.9913	0.036*	
C19	−0.0040 (2)	1.30890 (18)	0.94408 (14)	0.0305 (5)	
H19A	−0.0540	1.3448	0.9074	0.037*	
C20	−0.00485 (19)	1.19497 (17)	0.94788 (13)	0.0263 (5)	
H20A	−0.0547	1.1553	0.9133	0.032*	
C21	−0.02654 (17)	0.96925 (15)	1.09878 (11)	0.0203 (4)	
C22	−0.13214 (18)	1.03066 (16)	1.10886 (12)	0.0231 (5)	
H22A	−0.1524	1.0840	1.0711	0.028*	
C23	−0.2073 (2)	1.01308 (18)	1.17447 (13)	0.0277 (5)	
H23A	−0.2772	1.0551	1.1809	0.033*	
C24	−0.1788 (2)	0.93330 (19)	1.23050 (13)	0.0303 (5)	
H24A	−0.2297	0.9215	1.2744	0.036*	
C25	−0.0752 (2)	0.8714 (2)	1.22134 (13)	0.0319 (5)	
H25A	−0.0562	0.8173	1.2588	0.038*	
C26	0.0012 (2)	0.88959 (18)	1.15610 (12)	0.0281 (5)	
H26A	0.0716	0.8481	1.1506	0.034*	

### Atomic displacement parameters (Å^2^)

**Table:**

	*U*^11^	*U*^22^	*U*^33^	*U*^12^	*U*^13^	*U*^23^
Fe1	0.02261 (15)	0.01571 (14)	0.02675 (16)	−0.00025 (12)	0.00374 (13)	0.00058 (13)
P1	0.0201 (3)	0.0192 (3)	0.0186 (3)	0.0020 (2)	−0.0007 (2)	0.0016 (2)
O1	0.0219 (7)	0.0174 (7)	0.0297 (8)	−0.0010 (6)	0.0016 (6)	0.0015 (6)
C1	0.0283 (11)	0.0154 (10)	0.0378 (12)	0.0046 (9)	0.0035 (11)	0.0019 (10)
C2	0.0387 (14)	0.0162 (11)	0.0525 (15)	0.0022 (10)	0.0012 (12)	−0.0069 (10)
C3	0.0332 (14)	0.0176 (12)	0.103 (3)	−0.0032 (10)	0.0143 (17)	0.0083 (16)
C4	0.066 (2)	0.0258 (13)	0.0637 (19)	0.0156 (12)	0.0410 (16)	0.0200 (13)
C5	0.0570 (16)	0.0207 (11)	0.0343 (13)	0.0130 (11)	0.0039 (13)	0.0061 (10)
C6	0.0184 (10)	0.0137 (9)	0.0212 (10)	0.0026 (8)	0.0010 (8)	0.0027 (8)
C7	0.0203 (10)	0.0186 (10)	0.0240 (11)	0.0018 (8)	0.0014 (9)	0.0005 (8)
C8	0.0229 (11)	0.0258 (11)	0.0292 (12)	−0.0001 (10)	−0.0050 (10)	0.0000 (10)
C9	0.0275 (11)	0.0218 (10)	0.0196 (10)	0.0000 (9)	−0.0023 (9)	−0.0007 (8)
C10	0.0247 (11)	0.0148 (10)	0.0198 (10)	0.0011 (9)	0.0010 (9)	0.0004 (8)
C11	0.0243 (11)	0.0159 (10)	0.0209 (10)	0.0003 (9)	−0.0016 (9)	0.0005 (8)
C12	0.0309 (12)	0.0322 (12)	0.0287 (12)	0.0012 (10)	0.0077 (10)	−0.0052 (10)
C13	0.0230 (11)	0.0258 (12)	0.0386 (13)	−0.0053 (9)	0.0007 (11)	0.0006 (10)
C14	0.0289 (12)	0.0259 (12)	0.0454 (14)	−0.0053 (10)	0.0065 (11)	−0.0037 (11)
C15	0.0182 (11)	0.0198 (10)	0.0188 (10)	−0.0007 (8)	0.0048 (8)	−0.0003 (8)
C16	0.0269 (11)	0.0293 (12)	0.0198 (10)	−0.0029 (9)	−0.0046 (9)	0.0015 (9)
C17	0.0350 (13)	0.0291 (12)	0.0282 (12)	−0.0125 (10)	−0.0001 (11)	−0.0055 (10)
C18	0.0364 (14)	0.0188 (11)	0.0349 (13)	−0.0005 (9)	0.0095 (11)	0.0023 (10)
C19	0.0310 (12)	0.0240 (12)	0.0366 (12)	0.0028 (9)	−0.0037 (11)	0.0091 (10)
C20	0.0267 (11)	0.0229 (11)	0.0292 (11)	−0.0015 (9)	−0.0055 (10)	0.0005 (9)
C21	0.0235 (10)	0.0193 (10)	0.0181 (9)	−0.0017 (8)	−0.0012 (10)	0.0015 (8)
C22	0.0309 (12)	0.0192 (10)	0.0190 (10)	0.0000 (9)	−0.0003 (9)	0.0004 (8)
C23	0.0291 (12)	0.0276 (12)	0.0264 (11)	−0.0029 (10)	0.0045 (10)	−0.0071 (10)
C24	0.0346 (13)	0.0367 (13)	0.0196 (11)	−0.0114 (11)	0.0040 (10)	−0.0014 (10)
C25	0.0382 (14)	0.0332 (13)	0.0243 (12)	−0.0045 (11)	−0.0031 (10)	0.0095 (10)
C26	0.0313 (12)	0.0285 (12)	0.0245 (11)	0.0005 (10)	−0.0039 (10)	0.0038 (9)

### Geometric parameters (Å, °)

**Table:**

Fe1—C10	2.025 (2)	C11—C12	1.518 (3)
Fe1—C1	2.038 (2)	C11—H11A	0.9800
Fe1—C9	2.041 (2)	C12—H12A	0.9600
Fe1—C6	2.041 (2)	C12—H12B	0.9600
Fe1—C4	2.042 (2)	C12—H12C	0.9600
Fe1—C8	2.045 (2)	C13—C14	1.505 (3)
Fe1—C7	2.046 (2)	C13—H13A	0.9700
Fe1—C2	2.047 (2)	C13—H13B	0.9700
Fe1—C5	2.047 (2)	C14—H14A	0.9600
Fe1—C3	2.050 (2)	C14—H14B	0.9600
P1—C6	1.816 (2)	C14—H14C	0.9600
P1—C21	1.838 (2)	C15—C20	1.381 (3)
P1—C15	1.844 (2)	C15—C16	1.392 (3)
O1—C13	1.424 (3)	C16—C17	1.390 (3)
O1—C11	1.434 (2)	C16—H16A	0.9300
C1—C2	1.411 (3)	C17—C18	1.376 (3)
C1—C5	1.416 (3)	C17—H17A	0.9300
C1—H1A	0.9800	C18—C19	1.375 (3)
C2—C3	1.415 (4)	C18—H18A	0.9300
C2—H2A	0.9800	C19—C20	1.390 (3)
C3—C4	1.412 (4)	C19—H19A	0.9300
C3—H3A	0.9800	C20—H20A	0.9300
C4—C5	1.410 (4)	C21—C22	1.392 (3)
C4—H4A	0.9800	C21—C26	1.393 (3)
C5—H5A	0.9800	C22—C23	1.384 (3)
C6—C10	1.435 (3)	C22—H22A	0.9300
C6—C7	1.439 (3)	C23—C24	1.382 (3)
C7—C8	1.428 (3)	C23—H23A	0.9300
C7—H7A	0.9800	C24—C25	1.376 (3)
C8—C9	1.419 (3)	C24—H24A	0.9300
C8—H8A	0.9800	C25—C26	1.388 (3)
C9—C10	1.418 (3)	C25—H25A	0.9300
C9—H9A	0.9800	C26—H26A	0.9300
C10—C11	1.507 (3)		
			
C10—Fe1—C1	106.17 (9)	C8—C7—C6	108.05 (18)
C10—Fe1—C9	40.83 (8)	C8—C7—Fe1	69.55 (12)
C1—Fe1—C9	120.74 (9)	C6—C7—Fe1	69.22 (11)
C10—Fe1—C6	41.32 (7)	C8—C7—H7A	126.0
C1—Fe1—C6	123.26 (8)	C6—C7—H7A	126.0
C9—Fe1—C6	68.99 (8)	Fe1—C7—H7A	126.0
C10—Fe1—C4	158.10 (11)	C9—C8—C7	107.99 (18)
C1—Fe1—C4	67.90 (9)	C9—C8—Fe1	69.49 (12)
C9—Fe1—C4	160.50 (11)	C7—C8—Fe1	69.60 (12)
C6—Fe1—C4	122.93 (10)	C9—C8—H8A	126.0
C10—Fe1—C8	68.94 (9)	C7—C8—H8A	126.0
C1—Fe1—C8	156.45 (9)	Fe1—C8—H8A	126.0
C9—Fe1—C8	40.66 (8)	C10—C9—C8	108.57 (18)
C6—Fe1—C8	69.17 (8)	C10—C9—Fe1	69.00 (11)
C4—Fe1—C8	124.97 (10)	C8—C9—Fe1	69.86 (12)
C10—Fe1—C7	69.21 (8)	C10—C9—H9A	125.7
C1—Fe1—C7	160.92 (8)	C8—C9—H9A	125.7
C9—Fe1—C7	68.61 (8)	Fe1—C9—H9A	125.7
C6—Fe1—C7	41.22 (8)	C9—C10—C6	108.25 (18)
C4—Fe1—C7	109.11 (9)	C9—C10—C11	128.47 (18)
C8—Fe1—C7	40.85 (8)	C6—C10—C11	123.28 (18)
C10—Fe1—C2	122.21 (9)	C9—C10—Fe1	70.17 (12)
C1—Fe1—C2	40.41 (9)	C6—C10—Fe1	69.95 (11)
C9—Fe1—C2	106.50 (9)	C11—C10—Fe1	126.19 (14)
C6—Fe1—C2	159.33 (9)	O1—C11—C10	106.40 (16)
C4—Fe1—C2	67.84 (10)	O1—C11—C12	110.14 (17)
C8—Fe1—C2	121.37 (10)	C10—C11—C12	114.32 (17)
C7—Fe1—C2	157.70 (9)	O1—C11—H11A	108.6
C10—Fe1—C5	121.51 (10)	C10—C11—H11A	108.6
C1—Fe1—C5	40.56 (9)	C12—C11—H11A	108.6
C9—Fe1—C5	156.82 (9)	C11—C12—H12A	109.5
C6—Fe1—C5	107.69 (9)	C11—C12—H12B	109.5
C4—Fe1—C5	40.35 (11)	H12A—C12—H12B	109.5
C8—Fe1—C5	161.50 (9)	C11—C12—H12C	109.5
C7—Fe1—C5	124.93 (9)	H12A—C12—H12C	109.5
C2—Fe1—C5	68.07 (10)	H12B—C12—H12C	109.5
C10—Fe1—C3	158.99 (11)	O1—C13—C14	108.08 (18)
C1—Fe1—C3	68.00 (9)	O1—C13—H13A	110.1
C9—Fe1—C3	123.33 (12)	C14—C13—H13A	110.1
C6—Fe1—C3	158.72 (10)	O1—C13—H13B	110.1
C4—Fe1—C3	40.39 (12)	C14—C13—H13B	110.1
C8—Fe1—C3	107.86 (10)	H13A—C13—H13B	108.4
C7—Fe1—C3	122.83 (10)	C13—C14—H14A	109.5
C2—Fe1—C3	40.40 (10)	C13—C14—H14B	109.5
C5—Fe1—C3	68.07 (11)	H14A—C14—H14B	109.5
C6—P1—C21	101.20 (9)	C13—C14—H14C	109.5
C6—P1—C15	102.97 (9)	H14A—C14—H14C	109.5
C21—P1—C15	100.13 (8)	H14B—C14—H14C	109.5
C13—O1—C11	113.47 (16)	C20—C15—C16	118.39 (19)
C2—C1—C5	108.3 (2)	C20—C15—P1	125.17 (15)
C2—C1—Fe1	70.13 (13)	C16—C15—P1	116.40 (15)
C5—C1—Fe1	70.05 (12)	C17—C16—C15	120.8 (2)
C2—C1—H1A	125.9	C17—C16—H16A	119.6
C5—C1—H1A	125.9	C15—C16—H16A	119.6
Fe1—C1—H1A	125.9	C18—C17—C16	120.0 (2)
C1—C2—C3	108.0 (2)	C18—C17—H17A	120.0
C1—C2—Fe1	69.47 (12)	C16—C17—H17A	120.0
C3—C2—Fe1	69.90 (13)	C19—C18—C17	119.5 (2)
C1—C2—H2A	126.0	C19—C18—H18A	120.2
C3—C2—H2A	126.0	C17—C18—H18A	120.2
Fe1—C2—H2A	126.0	C18—C19—C20	120.6 (2)
C4—C3—C2	107.6 (2)	C18—C19—H19A	119.7
C4—C3—Fe1	69.49 (14)	C20—C19—H19A	119.7
C2—C3—Fe1	69.70 (13)	C15—C20—C19	120.57 (19)
C4—C3—H3A	126.2	C15—C20—H20A	119.7
C2—C3—H3A	126.2	C19—C20—H20A	119.7
Fe1—C3—H3A	126.2	C22—C21—C26	118.38 (19)
C5—C4—C3	108.6 (2)	C22—C21—P1	122.46 (15)
C5—C4—Fe1	70.04 (14)	C26—C21—P1	119.16 (15)
C3—C4—Fe1	70.12 (15)	C23—C22—C21	120.66 (19)
C5—C4—H4A	125.7	C23—C22—H22A	119.7
C3—C4—H4A	125.7	C21—C22—H22A	119.7
Fe1—C4—H4A	125.7	C24—C23—C22	120.2 (2)
C4—C5—C1	107.4 (2)	C24—C23—H23A	119.9
C4—C5—Fe1	69.61 (15)	C22—C23—H23A	119.9
C1—C5—Fe1	69.39 (13)	C25—C24—C23	120.0 (2)
C4—C5—H5A	126.3	C25—C24—H24A	120.0
C1—C5—H5A	126.3	C23—C24—H24A	120.0
Fe1—C5—H5A	126.3	C24—C25—C26	120.0 (2)
C10—C6—C7	107.14 (18)	C24—C25—H25A	120.0
C10—C6—P1	122.87 (15)	C26—C25—H25A	120.0
C7—C6—P1	129.82 (15)	C25—C26—C21	120.8 (2)
C10—C6—Fe1	68.73 (11)	C25—C26—H26A	119.6
C7—C6—Fe1	69.57 (11)	C21—C26—H26A	119.6
P1—C6—Fe1	123.06 (10)		
			
C10—Fe1—C1—C2	−121.03 (14)	Fe1—C6—C7—C8	−58.89 (14)
C9—Fe1—C1—C2	−79.11 (16)	C10—C6—C7—Fe1	58.67 (13)
C6—Fe1—C1—C2	−162.88 (13)	P1—C6—C7—Fe1	−116.57 (17)
C4—Fe1—C1—C2	81.32 (16)	C10—Fe1—C7—C8	81.47 (13)
C8—Fe1—C1—C2	−46.7 (3)	C1—Fe1—C7—C8	160.7 (2)
C7—Fe1—C1—C2	166.0 (2)	C9—Fe1—C7—C8	37.56 (12)
C5—Fe1—C1—C2	119.1 (2)	C6—Fe1—C7—C8	119.68 (17)
C3—Fe1—C1—C2	37.59 (16)	C4—Fe1—C7—C8	−121.79 (15)
C10—Fe1—C1—C5	119.87 (14)	C2—Fe1—C7—C8	−43.7 (3)
C9—Fe1—C1—C5	161.79 (14)	C5—Fe1—C7—C8	−163.90 (13)
C6—Fe1—C1—C5	78.03 (16)	C3—Fe1—C7—C8	−79.16 (17)
C4—Fe1—C1—C5	−37.78 (16)	C10—Fe1—C7—C6	−38.21 (11)
C8—Fe1—C1—C5	−165.8 (2)	C1—Fe1—C7—C6	41.0 (3)
C7—Fe1—C1—C5	46.9 (3)	C9—Fe1—C7—C6	−82.12 (12)
C2—Fe1—C1—C5	−119.1 (2)	C4—Fe1—C7—C6	118.53 (14)
C3—Fe1—C1—C5	−81.51 (17)	C8—Fe1—C7—C6	−119.68 (17)
C5—C1—C2—C3	0.4 (2)	C2—Fe1—C7—C6	−163.4 (2)
Fe1—C1—C2—C3	−59.49 (16)	C5—Fe1—C7—C6	76.41 (15)
C5—C1—C2—Fe1	59.89 (14)	C3—Fe1—C7—C6	161.16 (15)
C10—Fe1—C2—C1	76.58 (16)	C6—C7—C8—C9	−0.4 (2)
C9—Fe1—C2—C1	118.33 (14)	Fe1—C7—C8—C9	−59.09 (14)
C6—Fe1—C2—C1	44.2 (3)	C6—C7—C8—Fe1	58.68 (13)
C4—Fe1—C2—C1	−81.47 (16)	C10—Fe1—C8—C9	37.24 (12)
C8—Fe1—C2—C1	160.08 (13)	C1—Fe1—C8—C9	−44.9 (3)
C7—Fe1—C2—C1	−167.9 (2)	C6—Fe1—C8—C9	81.63 (13)
C5—Fe1—C2—C1	−37.77 (14)	C4—Fe1—C8—C9	−162.03 (14)
C3—Fe1—C2—C1	−119.2 (2)	C7—Fe1—C8—C9	119.40 (17)
C10—Fe1—C2—C3	−164.18 (16)	C2—Fe1—C8—C9	−78.50 (15)
C1—Fe1—C2—C3	119.2 (2)	C5—Fe1—C8—C9	165.2 (3)
C9—Fe1—C2—C3	−122.42 (17)	C3—Fe1—C8—C9	−120.72 (15)
C6—Fe1—C2—C3	163.5 (3)	C10—Fe1—C8—C7	−82.16 (12)
C4—Fe1—C2—C3	37.78 (18)	C1—Fe1—C8—C7	−164.33 (19)
C8—Fe1—C2—C3	−80.68 (19)	C9—Fe1—C8—C7	−119.40 (17)
C7—Fe1—C2—C3	−48.7 (3)	C6—Fe1—C8—C7	−37.77 (12)
C5—Fe1—C2—C3	81.48 (18)	C4—Fe1—C8—C7	78.57 (16)
C1—C2—C3—C4	−0.2 (3)	C2—Fe1—C8—C7	162.10 (12)
Fe1—C2—C3—C4	−59.38 (16)	C5—Fe1—C8—C7	45.8 (3)
C1—C2—C3—Fe1	59.22 (15)	C3—Fe1—C8—C7	119.88 (15)
C10—Fe1—C3—C4	158.9 (2)	C7—C8—C9—C10	0.9 (2)
C1—Fe1—C3—C4	81.29 (16)	Fe1—C8—C9—C10	−58.27 (14)
C9—Fe1—C3—C4	−165.50 (15)	C7—C8—C9—Fe1	59.16 (14)
C6—Fe1—C3—C4	−45.1 (4)	C1—Fe1—C9—C10	−78.90 (14)
C8—Fe1—C3—C4	−123.40 (15)	C6—Fe1—C9—C10	38.18 (11)
C7—Fe1—C3—C4	−80.96 (18)	C4—Fe1—C9—C10	169.5 (3)
C2—Fe1—C3—C4	118.9 (2)	C8—Fe1—C9—C10	120.27 (18)
C5—Fe1—C3—C4	37.39 (15)	C7—Fe1—C9—C10	82.54 (13)
C10—Fe1—C3—C2	40.1 (3)	C2—Fe1—C9—C10	−120.49 (13)
C1—Fe1—C3—C2	−37.59 (15)	C5—Fe1—C9—C10	−47.8 (3)
C9—Fe1—C3—C2	75.63 (18)	C3—Fe1—C9—C10	−161.40 (12)
C6—Fe1—C3—C2	−163.9 (2)	C10—Fe1—C9—C8	−120.27 (18)
C4—Fe1—C3—C2	−118.9 (2)	C1—Fe1—C9—C8	160.83 (12)
C8—Fe1—C3—C2	117.72 (16)	C6—Fe1—C9—C8	−82.09 (13)
C7—Fe1—C3—C2	160.17 (14)	C4—Fe1—C9—C8	49.2 (3)
C5—Fe1—C3—C2	−81.48 (16)	C7—Fe1—C9—C8	−37.73 (12)
C2—C3—C4—C5	−0.1 (3)	C2—Fe1—C9—C8	119.24 (13)
Fe1—C3—C4—C5	−59.65 (16)	C5—Fe1—C9—C8	−168.1 (2)
C2—C3—C4—Fe1	59.51 (16)	C3—Fe1—C9—C8	78.33 (16)
C10—Fe1—C4—C5	−40.2 (3)	C8—C9—C10—C6	−1.0 (2)
C1—Fe1—C4—C5	37.97 (14)	Fe1—C9—C10—C6	−59.82 (13)
C9—Fe1—C4—C5	158.4 (2)	C8—C9—C10—C11	179.80 (19)
C6—Fe1—C4—C5	−78.28 (16)	Fe1—C9—C10—C11	121.0 (2)
C8—Fe1—C4—C5	−164.60 (13)	C8—C9—C10—Fe1	58.80 (15)
C7—Fe1—C4—C5	−121.88 (14)	C7—C6—C10—C9	0.8 (2)
C2—Fe1—C4—C5	81.75 (15)	P1—C6—C10—C9	176.41 (14)
C3—Fe1—C4—C5	119.5 (2)	Fe1—C6—C10—C9	59.96 (14)
C10—Fe1—C4—C3	−159.8 (2)	C7—C6—C10—C11	179.99 (17)
C1—Fe1—C4—C3	−81.58 (15)	P1—C6—C10—C11	−4.4 (3)
C9—Fe1—C4—C3	38.8 (3)	Fe1—C6—C10—C11	−120.81 (18)
C6—Fe1—C4—C3	162.18 (14)	C7—C6—C10—Fe1	−59.20 (13)
C8—Fe1—C4—C3	75.85 (17)	P1—C6—C10—Fe1	116.45 (14)
C7—Fe1—C4—C3	118.57 (15)	C1—Fe1—C10—C9	118.58 (13)
C2—Fe1—C4—C3	−37.80 (15)	C6—Fe1—C10—C9	−119.07 (17)
C5—Fe1—C4—C3	−119.5 (2)	C4—Fe1—C10—C9	−170.6 (2)
C3—C4—C5—C1	0.4 (3)	C8—Fe1—C10—C9	−37.08 (12)
Fe1—C4—C5—C1	−59.31 (15)	C7—Fe1—C10—C9	−80.95 (13)
C3—C4—C5—Fe1	59.71 (17)	C2—Fe1—C10—C9	77.55 (15)
C2—C1—C5—C4	−0.5 (2)	C5—Fe1—C10—C9	159.99 (13)
Fe1—C1—C5—C4	59.45 (16)	C3—Fe1—C10—C9	48.0 (3)
C2—C1—C5—Fe1	−59.94 (15)	C1—Fe1—C10—C6	−122.34 (12)
C10—Fe1—C5—C4	163.58 (15)	C9—Fe1—C10—C6	119.07 (17)
C1—Fe1—C5—C4	−118.8 (2)	C4—Fe1—C10—C6	−51.5 (3)
C9—Fe1—C5—C4	−161.8 (2)	C8—Fe1—C10—C6	81.99 (12)
C6—Fe1—C5—C4	120.39 (16)	C7—Fe1—C10—C6	38.12 (11)
C8—Fe1—C5—C4	43.3 (4)	C2—Fe1—C10—C6	−163.37 (12)
C7—Fe1—C5—C4	78.15 (18)	C5—Fe1—C10—C6	−80.93 (14)
C2—Fe1—C5—C4	−81.13 (16)	C3—Fe1—C10—C6	167.1 (2)
C3—Fe1—C5—C4	−37.42 (16)	C1—Fe1—C10—C11	−5.16 (19)
C10—Fe1—C5—C1	−77.66 (15)	C9—Fe1—C10—C11	−123.7 (2)
C9—Fe1—C5—C1	−43.0 (3)	C6—Fe1—C10—C11	117.2 (2)
C6—Fe1—C5—C1	−120.84 (13)	C4—Fe1—C10—C11	65.6 (3)
C4—Fe1—C5—C1	118.8 (2)	C8—Fe1—C10—C11	−160.82 (19)
C8—Fe1—C5—C1	162.1 (2)	C7—Fe1—C10—C11	155.30 (19)
C7—Fe1—C5—C1	−163.08 (12)	C2—Fe1—C10—C11	−46.2 (2)
C2—Fe1—C5—C1	37.63 (13)	C5—Fe1—C10—C11	36.2 (2)
C3—Fe1—C5—C1	81.34 (15)	C3—Fe1—C10—C11	−75.7 (3)
C21—P1—C6—C10	−163.25 (16)	C13—O1—C11—C10	146.74 (17)
C15—P1—C6—C10	93.47 (17)	C13—O1—C11—C12	−88.9 (2)
C21—P1—C6—C7	11.3 (2)	C9—C10—C11—O1	111.4 (2)
C15—P1—C6—C7	−91.94 (19)	C6—C10—C11—O1	−67.6 (2)
C21—P1—C6—Fe1	−78.69 (12)	Fe1—C10—C11—O1	−156.03 (13)
C15—P1—C6—Fe1	178.04 (11)	C9—C10—C11—C12	−10.4 (3)
C1—Fe1—C6—C10	76.02 (14)	C6—C10—C11—C12	170.58 (18)
C9—Fe1—C6—C10	−37.74 (12)	Fe1—C10—C11—C12	82.2 (2)
C4—Fe1—C6—C10	159.63 (14)	C11—O1—C13—C14	179.14 (17)
C8—Fe1—C6—C10	−81.41 (12)	C6—P1—C15—C20	5.9 (2)
C7—Fe1—C6—C10	−118.85 (17)	C21—P1—C15—C20	−98.16 (18)
C2—Fe1—C6—C10	43.3 (3)	C6—P1—C15—C16	−171.67 (15)
C5—Fe1—C6—C10	117.91 (13)	C21—P1—C15—C16	84.23 (16)
C3—Fe1—C6—C10	−167.2 (3)	C20—C15—C16—C17	1.5 (3)
C10—Fe1—C6—C7	118.85 (16)	P1—C15—C16—C17	179.30 (17)
C1—Fe1—C6—C7	−165.12 (12)	C15—C16—C17—C18	−0.6 (3)
C9—Fe1—C6—C7	81.11 (12)	C16—C17—C18—C19	−0.3 (3)
C4—Fe1—C6—C7	−81.52 (15)	C17—C18—C19—C20	0.2 (3)
C8—Fe1—C6—C7	37.44 (12)	C16—C15—C20—C19	−1.6 (3)
C2—Fe1—C6—C7	162.1 (2)	P1—C15—C20—C19	−179.18 (17)
C5—Fe1—C6—C7	−123.24 (13)	C18—C19—C20—C15	0.8 (3)
C3—Fe1—C6—C7	−48.4 (3)	C6—P1—C21—C22	−68.60 (17)
C10—Fe1—C6—P1	−116.20 (17)	C15—P1—C21—C22	36.93 (18)
C1—Fe1—C6—P1	−40.17 (16)	C6—P1—C21—C26	111.64 (17)
C9—Fe1—C6—P1	−153.94 (14)	C15—P1—C21—C26	−142.83 (16)
C4—Fe1—C6—P1	43.43 (16)	C26—C21—C22—C23	0.3 (3)
C8—Fe1—C6—P1	162.39 (14)	P1—C21—C22—C23	−179.51 (16)
C7—Fe1—C6—P1	124.95 (17)	C21—C22—C23—C24	−0.6 (3)
C2—Fe1—C6—P1	−72.9 (3)	C22—C23—C24—C25	0.3 (3)
C5—Fe1—C6—P1	1.71 (14)	C23—C24—C25—C26	0.4 (3)
C3—Fe1—C6—P1	76.6 (3)	C24—C25—C26—C21	−0.8 (3)
C10—C6—C7—C8	−0.2 (2)	C22—C21—C26—C25	0.5 (3)
P1—C6—C7—C8	−175.46 (15)	P1—C21—C26—C25	−179.76 (17)
